# Estimating the impact of changes in weight and BMI on EQ-5D-3L: a longitudinal analysis of a behavioural group-based weight loss intervention

**DOI:** 10.1007/s11136-022-03178-z

**Published:** 2022-07-07

**Authors:** Penny Breeze, Laura A. Gray, Chloe Thomas, Sarah E. Bates, Alan Brennan

**Affiliations:** School of Health and Related Research, University of Sheffield, Regent Court, 30 Regent Street, Sheffield, S1 4DA UK

**Keywords:** Obesity, Health economics, Quality of life, Weight-loss

## Abstract

**Purpose:**

To estimate the association between changes in BMI and changes in Health-Related Quality of Life (EQ-5D-3L).

**Methods:**

The WRAP trial was a multicentre, randomised controlled trial with parallel design and recruited 1267 adults (BMI ≥ 28 kg/m^2^). Participants were allocated to Brief Intervention, a Commercial weight management Programme (WW, formerly Weight Watchers) for 12 weeks, or the same Programme for 52 weeks. Participants were assessed at 0, 3, 12, 24, and 60 months. We analysed the relationship between BMI and EQ-5D-3L, adjusting for age and comorbidities, using a fixed effects model. Test for attrition, model specification and missing data were conducted. Secondary analyses investigated a non-symmetric gradient for weight loss vs. regain.

**Results:**

A unit increase in BMI was associated with a − 0.011 (95% CI − 0.01546, − 0.00877) change in EQ-5D-3L. A unit change in BMI between periods of observation was associated with − 0.016 017 (95% CI − 0.0077009, − 0.025086) change in EQ-5D-3L. The negative association was reduced during weight loss, as opposed to weight gain, but the difference was not statistically significant.

**Conclusions:**

We have identified a strong and statistically significant negative relationship between BMI changes and HRQoL. These estimates could be used in economic evaluations of weight loss interventions to inform policymaking.

**Clinical trial registration:**

This trial was registered with Current Controlled Trials, number ISRCTN82857232.

**Supplementary Information:**

The online version contains supplementary material available at 10.1007/s11136-022-03178-z.

## Plain English summary

It is useful to understand if weight loss interventions offer value for money to healthcare providers. It is important to consider first, whether weight loss will lead to short-term improvements in quality of life, and second, whether this improvement in quality of life is maintained. This study uses a large sample of data from individuals who wanted to lose weight and received different weight loss interventions. Their weight, BMI and quality of life were measured over 5 years. We look at the relationships between BMI and quality of life to investigate how weight loss and weight regain may be affecting people’s quality of life. We find that higher BMI is associated with poorer quality of life and weight loss was linked to improvements in quality of life. We did not find enough evidence to suggest that weight loss may have less effect on quality of life, than weight regain. Our estimates can be used to describe the relationship between BMI and quality of life when evaluating the value of weight loss interventions.

## Introduction

Obesity increases the risk of chronic health conditions, including type-2 diabetes and cardiovascular disease (CVD), and consequently will impact Health-Related Quality of Life (HRQoL) as health deteriorates. It is less clear whether weight loss and weight gain, in the absence of disease complications, leads to changes in HRQoL. This association is particularly important when evaluating the cost-utility of weight loss interventions, where estimated Quality-Adjusted Life Year (QALY) gains may include HRQoL improvements due to weight loss.

The EuroQol 5-Dimension 3-level (EQ-5D-3L) questionnaire is a standardised measure of HRQoL to provide a simple, generic questionnaire for use in clinical and economic appraisal and population health surveys. The EQ-5D-3L consists of a single question in each of five domains: Mobility, Self-Care, Usual activity, Pain/Discomfort, and Anxiety/Depression. The EQ-5D-3L has been accompanied by value sets to derived utilities that can be used in the calculation of QALYs [1]. Cross-sectional analyses of general population samples illustrate that people with obesity report lower EQ-5D-3L compared to those with Body Mass Index (BMI) < 25 kg/m^2^ after adjustment for co-morbid conditions [2]. However, cross-sectional analyses are more likely to be influenced by unobservable confounding factors related to both BMI and HRQoL [3]. Evidence from longitudinal studies and randomised controlled trials (RCTs) offers inconsistent findings and suggests complex patterns in the relationship between BMI and HRQoL. Overall, studies from longitudinal analyses of population cohort studies have identified significant associations between weight (or BMI) changes and HRQoL, particularly on the physical scale [4–8]. Three reviews of Randomised Controlled Trials (RCTs) for weight loss interventions suggest a weak or inconsistent association [9–11], particularly for non-surgical interventions [9, 11]. However, there are examples of non-surgical trials demonstrating a strong association between weight loss and BMI change [12, 13].

The strength of association between BMI changes and HRQoL have been found to be asymmetrical between weight loss and weight gain. Longitudinal analyses of EQ-5D-3L indicate that weight gain was significantly associated with reductions in EQ-5D-3L, whereas a weaker or no association was observed for weight loss [4]. This may explain why findings from weight loss RCTs, with shorter follow-up, indicate a weaker association between weight changes and HRQoL, compared with cohort studies. Alternatively, the association between weight loss and HRQoL may be weakened by the effects of weight loss due to poor health, which is challenging to control for in cohort studies, but less likely to dominate the effects in population intending to lose weight.

Previous reviews and studies of the effects of weight loss on HRQoL have focussed on the relationships between the Short Form-36 (SF-36) physical and mental health outcomes [6–10]. Although widely reported, the associations between BMI and SF-36 score cannot be directly used in cost–utility analyses of new interventions. Cost–utility analyses require a single preferences-based index for HRQoL that can be used to generate QALYs. The EQ-5D-3L is a commonly used preference-based measure of HRQoL recommended by NICE in appraisal of new health technologies [14].

The aim of this analysis is to estimate the impact of BMI, on EQ-5D-3L in a population with BMI > 28 kg/m^2^ using 5 years of follow-up of the Weight Loss Referrals in Primary care (WRAP) trial [15]. We also aimed to investigate if the effects of BMI on EQ-5D-3L are symmetrical during weight loss and weight gain.

## Method

### The data

We performed a secondary data analysis of RCT data to establish a longitudinal association between BMI and HRQoL. The WRAP trial is a multi-centre, non-blinded multi-arm UK-based RCT. The full protocol and earlier trial results have been reported elsewhere, including the 12 and 24 month change in EQ-5D-3L by randomised group [15]. Participants were recruited from 23 primary care practices in England (October 2012 to February 2014). This trial was registered with Current Controlled Trials, number ISRCTN82857232. Recruitment criteria included a BMI greater than 28 kg/m^2^ and aged 18 years or older. Exclusion criteria were planned or current pregnancy; previous or planned bariatric surgery; current participation in a structured, monitored weight-loss programme; participation in other research; eating disorders; and non-English speaking or special communication needs. Eligible individuals were identified from an electronic register. Participants were randomised to one of three arms: brief intervention, 12-weeks of a behavioural programme, or 52 weeks of a behavioural programme. The behavioural programme involved attendance at a local WW (formerly Weight Watchers) meeting once a week for the duration of the programme. WW is a widely available behavioural weight management and wellness commercial program that has been studied extensively [15, 16]. Participants allocated to the brief intervention were given a 32-page printed booklet by the British Heart Foundation of self-help weight-management strategies [17] and research staff read a scripted introduction that drew attention to each section of the booklet.

Data collection was originally scheduled at baseline and 3, 12, and 24 months. At 5 years an additional study data collection was conducted, with data collected for 69% of participants.

### Measurement of weight and Body Mass Index

All participants attended appointments at a study centre or General Practice (GP) at baseline, 3, 12 and 24 months. Height was measured to the nearest 0.1 cm using a stadiometer. Weight was measured to the nearest 0.1 kg. Participants who were unable or unwilling to attend a 12-month visit (primary outcome measurement) were asked to provide a self-measured weight. At 5 years, the majority of participants attended a measurement appointment at a study centre, with 17% and 11% of participants providing measurements from GP notes review or self-report, respectively.

### HRQoL measures

HRQoL is a multi-dimensional concept that measures perceived physical and mental health through domains related to physical, mental, emotional, and social functioning [18]. At baseline, 3 months, 12 months, 24 months and 5 years all participants completed the EQ-5D-3L questionnaire [1, 19], to measure quality of life. The EQ-5D-3L is designed to produce a HRQoL score that is preference-based and set between the values of 0 (death) and 1 (full health), however like many preference-based utility instruments, it produces scores that are deemed to be ‘worse than death’ and therefore have values of less than 0. This score when combined with life years can be used to generate QALYs for health economic evaluation.

### Comorbidity measures

Comorbid conditions and health events associated with obesity were collected within the trial. These comorbid conditions were included in our analysis to adjust the analysis for their effects on HRQoL. The data is not exhaustive of all health conditions, but includes health conditions associated with obesity.

Diagnosis with type-2 diabetes was indicated from GP record data recording a diabetes diagnosis, or diabetic medication. In addition, participants were classified as diabetic if they report Hba1c > 47 mmol/mol at a study visit. Clinical events related to coronary heart disease, peripheral vascular disease, stroke (including Transient Ischemic Attack), or cancer were recorded from GP records. Events relating to coronary heart disease, stroke and peripheral vascular disease were combined into a single cardiovascular disease category due to low incidence events.

Anti-depression medication, hypertension medication and statin use were collected in the trial from GP records. We use records of anti-depression medication as an indication of depression in our analyses due to the strong association between depression and EQ-5D-3L. Hypertension and statin use are less likely to be associated with quality of life so were not included in our primary analyses but were included in sensitivity analyses.

We considered several model specifications to include comorbidities including adding each comorbidity individually, together, with interaction terms and as an index of multi-morbidity combining diabetes, cancer, CVD and depression. Cases of anti-depression medication were added to the multimorbidity index. Missing data for anti-depression medication were assumed to indicate no depression for the multimorbidity index in order that the observations were included in the analysis.

### Statistical analysis

To explore the cross-sectional data, we report mean (Standard Deviation) EQ-5D-3L scores by BMI categories at baseline (28 kg/m^2^-30 kg/m^2^, 30 kg/m^2^-35 kg/m^2^, 35 kg/m^2^+). A one-way ANOVA assessed the statistical significance of differences in EQ-5D-3L across categories.

To explore unadjusted relationships between weight loss or weight gain and changes in EQ-5D-3L over the course of the RCT, we segmented the data into two phases. The first phase of the trial up to 12 months assessed the relationship between weight loss and changes in HRQoL. In the second phase of the trial, we assessed the effect of weight maintenance and weight regain on HRQoL. We summarise EQ-5D-3L between baseline and 3 months and baseline and 12 months by weight loss categories (weight increase, < 5% weight loss, ≥ 5% and < 10% weight loss, ≥ 10% weight loss). We explore the relationship between weight maintenance/regain and changes in EQ-5D-3L between 12 and 24 months and 12 months and 5 years by looking at the mean change in EQ-5D-3L for weight maintenance/regain categories (> 10% weight regain, > 5% and < 10% weight regain, < 5% weight regain, weight loss). The differences between EQ-5D-3L across categories was assessed using a one-way ANOVA.

We used regression techniques to adjust for covariates and unobserved heterogeneity within RCT participants. For our first regression analysis we estimated the impact of BMI on EQ-5D-3L using a generalised least squares fixed effects model. The fixed effects model specification was compared with a random effects model, and selected based on the Hausman test. The model specification is expressed as follows:

$${EQ5D}_{ij}={\beta }_{1}+{\beta }_{2}{BMI}_{ij}+\beta {X}_{ij}+{\upsilon }_{i}+{\epsilon }_{ij},$$

where EQ-5D-3L is estimated as a function of BMI for an individual i, and time j, and a vector of time-varying covariates ${X}_{ij}.$ The model includes a time-invariant individual specific term (${v}_{i}$) known as unobserved heterogeneity and, a time-variant error term (${\varepsilon }_{it}$). EQ-5D-3L responses are limited at 1 and -0.594, and unlikely to be normally distributed with a large cluster of observations at the maximum score of 1. A sensitivity analysis in which a random effects Tobit regression specification with limits at 1 and -0.594 is estimated to test the robustness of the results with an alternative specification. The Tobit model explicitly accounts for the limited nature of EQ-5D-3L distribution and does not allow predicted values of EQ-5D-3L above 1.

The model specifications estimate the EQ-5D-3L conditional on BMI, adjusted for potential confounders (age and comorbid conditions). Demographic variables that were time invariant cannot be retained in a fixed effects model specification. We report three model specifications, the first with covariates for BMI, age and age-squared but without including covariates for comorbidities (model specification 1), the second includes the covariates of the first with the addition of diabetes, CVD and cancer (model specification 2), and the third specification includes the covariates from the first but with an index of comorbid diabetes, cancer, CVD and depression (model specification 3). Interactions between comorbidities were considered, but did not substantially improve the model specification based on the Akaike Information Criterion [20] and Bayesian Information Criterion [21], so were not included in the final analysis. An alternative set of analyses in which weight, rather than BMI, was included as the main explanatory variable were investigated and the results are reported in the supplementary material.

Our second set of analyses investigated the relationships between changes in EQ-5D-3L between observations and changes in BMI, adjusting for baseline EQ-5D-3L and baseline BMI (model specification 4). In model specification 5 we test the impact of including an interaction term to stratify the analysis for periods of weight loss from weight regain to allow asymmetric effects. An ordinary least squares regression specification was used, with robust standard errors. The model specification can be expressed as follows:

$${\Delta EQ5D}_{ij}={\beta }_{1}+{\beta }_{2}\Delta {BMI}_{ij}+{\beta }_{3}loss*\Delta {BMI}_{ij}+\beta {X}_{i}+{\epsilon }_{ij},$$

where EQ-5D-3L is a function of BMI for individual i, and time j, an additional variable is included for negative changes in BMI to estimate an alternative slope for weight loss compared with weight gain. We include a vector of covariates ${X}_{ij}.$ Covariates included baseline EQ-5D-3L, and randomised group. The model includes a time-variant error term (${\varepsilon }_{it}$). We explored the impact of comorbidities index on the relationships between BMI and EQ-5D-3L in model specification 6. We report results in the supplementary material for an alternative set of analyses in which weight change, rather than BMI change is the main explanatory variable. Time periods between trial observations are not equally spaced (3 months, 9 months, 12 months and 36 months). We stratified the analysis by time period to observe the consistency in the relationship between BMI and EQ-5D-3L across trial periods, also reported in the supplementary material.

We used the Wooldridge test for attrition bias [22, 23]. No problems with attrition were identified so in our final model specifications we conducted complete case analysis (Tables S1 and S2). However, in the supplementary appendix we report regression specifications where only participants attending follow-up at 5 years are included and missing data for EQ-5D-3L, BMI, Index of Multiple Deprivation (IMD) and comorbidities are imputed using multiple imputation with chained equations (MICE). Missing data for age were imputed manually based on age reported at baseline. We estimate imputed variables conditional on baseline age, BMI, sex and randomised group. We imputed 40 datasets for the final regression analysis and assumed that BMI and EQ-5D-3L were normally distributed. All statistical analyses were conducted using STATA 15 [24].

## Results

**Fig. 1:**
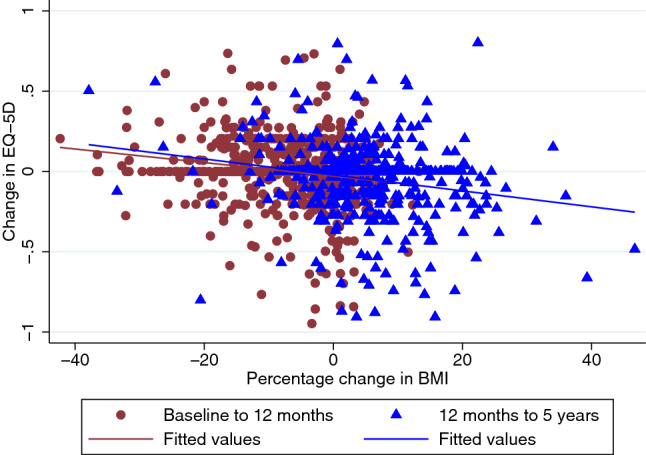
A scatter plot illustrating patterns of BMI change and EQ-5D over two periods of study follow-up from baseline to 12 months and 12 months to 5 years

Missing data and population characteristics at baseline, are detailed in Table 1. The population has more women, with more participants from the least deprived groups. Figure 1 illustrates negative correlation between change in weight and EQ-5D-3L and the pattern is consistent over time. Table 2 describes summary statistics for EQ-5D-3L throughout the trial stratified by BMI and weight gain/loss categories. The data suggests that the cross-sectional relationship between BMI and EQ-5D-3L is negative and significant with those in the highest BMI category reporting worse EQ-5D-3L. In the first 3 months of the trial, weight change was not significantly associated with changes in EQ-5D-3L. However, the changes in weight 12 months into the trial were significantly associated with EQ-5D-3L. EQ-5D-3L improvements were greatest in the group with the largest weight loss.

**Table 1:** Baseline characteristics of WRAP trial participants

Variable	Number in category	%	Missing data (%)
Women	859	67.80	0
University degree or higher	423	33.39	130 (12.26)
Economically active	767	60.54	32 (2.53)
IMD quintile			
1 (most deprived)	155	12.23	2 (0.16)
2	174	13.75	2 (0.16)
3	267	21.11	2 (0.16)
4	325	25.69	2 (0.16)
5 (least deprived)	344	27.19	2 (0.16)
Diabetes	165	13.02	137 (10.81)
CVD	2	0.16	0 (0)
Cancer	5	0.39	0 (0)
Depression	188	14.84	396 (31.25)
Hypertensive medication	347	27.39	396 (31.25)
Statin use	225	17.76	396 (31.25)
Treatment allocation			
Brief intervention	211	16.66	0
12 week WW	528	41.67	0
52 week WW	528	41.67	0

	Mean	SD	Missing data (%)
BMI (kg/m^2^)	34.54	5.127	0
Weight (kg)	96.16	17.052	0
EQ-5D-3L	0.788	0.252	58 (4.58)
Age	53.20	13.69	0

*IMD* Index of Multiple Deprivation small area measure of relative deprivation [31]; WW refers to the intervention arm of the trial (formerly Weight Watchers), *BMI* Body Mass Index

**Table 2:** Summary statistics for EQ-5D-3L by BMI category and changes in EQ-5D-3L between trial time-points stratified by % weight change

Group categories	Mean EQ-5D-3L at baseline stratified by BMI category			
	N	Mean	Standard deviation	F-statistic (*p* value)
All participants	1209	0.788	0.252	
28–30 kg/m^2^	184	0.846	0.208	19.66 (*p* < 0.000)
30–35 kg/m^2^	577	0.813	0.226	
≥ 35 kg/m^2^	448	0.731	0.288	

	Changes in EQ-5D-3L from baseline to 3 months			
% weight change from baseline to 3 months	N	Mean	Standard deviation	F-statistic (*p* value)
All participants	883	0.0047	0.1700	
> 10% weight gain	1	0.0710	0.0000	1.06 (0.3796)
5–10% weight gain	1	− 0.1920	0.0000	
< 5% weight gain	82	− 0.0149	0.1787	
< 5% weight loss	408	0.0052	0.1702	
5–10% weight loss	307	0.0015	0.1740	
> 10% weight loss	84	0.0346	0.1411	

	Changes in EQ-5D-3L from baseline to 12 months			
% weight change from baseline to 12 months	N	Mean	Standard deviation	F-statistic (*p* value)
All participants	722	− 0.0020	0.1945	
> 10% weight gain	3	− 0.2373	0.2527	5.83 (*p* < 0.000)
5–10% weight gain	19	− 0.0321	0.1294	
< 5% weight gain	120	− 0.0549	0.2233	
< 5% weight loss	227	− 0.0196	0.1960	
5–10% weight loss	150	0.0124	0.1538	
> 10% weight loss	203	0.0448	0.1946	

	Changes in EQ-5D-3L from 12 to 24 months			
% weight change from 12 to 24 months	N	Mean	Standard deviation	F-statistic (*p* value)
All participants	620	− 0.0135	0.1874	
> 10% weight gain	44	− 0.0026	0.2408	0.20 (0.962)
5–10% weight gain	116	− 0.0191	0.1635	
< 5% weight gain	291	− 0.0187	0.1909	
< 5% weight loss	129	− 0.0019	0.1861	
5–10% weight loss	26	− 0.0062	0.1919	
> 10% weight loss	14	− 0.0125	0.1241	

	Changes in EQ-5D-3L from 12 months to 5 years			
% weight change from 12 months to 5 years	N	Mean	Standard deviation	F-statistic (*p* value)
All participants	465	− 0.0494	0.2343	
> 10% weight gain	112	− 0.1068	0.2453	3.86 (0.002)
5–10% weight gain	108	− 0.0639	0.2394	
< 5% weight gain	123	− 0.0332	0.2364	
< 5% weight loss	72	− 0.0282	0.0180	
5–10% weight loss	34	− 0.0018	0.1548	
> 10% weight loss	16	0.1266	0.3387	

This table demonstrates the average change in EQ-5D-3L across periods of observation in the trial. The study sample has been segmented by percentage change in weight to illustrate how change in EQ-5D differs for those experiencing weight loss and weight gain. The F statistics are generated from One Way Analysis of Variance

Weight changes between 12 and 24 months were not strongly associated with changes in EQ-5D-3L. Whereas, weight changes from 12 to 60 months (5 years), were strongly and negatively associated with changes in EQ-5D-3L, with those participants experiencing the greatest weight gain also having the largest reduction in EQ-5D-3L. There is some evidence from this analysis that the relationships between weight loss and weight gain and EQ-5D-3L are different. The average change in EQ-5D-3L between baseline and 12 months for the group with more than 10% weight loss is approximately half the size of the change in EQ-5D-3L between 12 and 60 months for the group with more than 10% weight gain.

The results of our primary regression analysis looking at the longitudinal relationship between BMI and EQ-5D-3L are reported in Table 3. In model specification 1, with no comorbid adjustment, a unit increase in BMI reduced EQ-5D-3L by 0.0107 and the association is statistically significant. Age has a negative and significant relationship with EQ-5D-3L, and the association becomes weaker in older age. In models specifications 2 and 3 where comorbid conditions have been included and most comorbidities are not significantly related to changes in EQ-5D-3L. Their inclusion does not substantially alter the coefficient size and effect direction for BMI. Depression reduced HRQoL by 0.044 and the coefficient was significant. The results of the regression using weight, rather than BMI, to explain variation in EQ-5D- finds a similar relationship between weight and EQ-5D-3L (Table S3). We also report analyses with other medications (Table S4), multiple imputation (Table S5), only participants attending 5 year follow-up (Table S6), and random effects Tobit model (Table S7) in the supplementary material. The results are very similar to the main analysis and do not suggest any problems with the main analysis. Depression and the multi-morbidity index show statistical significance in the multiple imputation analysis.

**Table 3:** Longitudinal regression estimates predicting EQ-5D-3L conditional on BMI and other associated factors

Variable	Model specification 1: no co-morbidities		Model specification 2: independent covariate		Model specification 3: multi-morbidity score	
	Coefficient estimate	Standard error	Coefficient estimate	Standard error	Coefficient estimate	Standard error
BMI	− 0.0107	0.0016 ***	− 0.0121	0.0020***	− 0.0111	0.0018***
Age	− 0.0206	0.0082 **	− 0.0247	0.0112**	− 0.0264	0.0086***
Age-squared	0.0001	0.0001	0.0001	0.0001	0.0001	0.0001
Diabetes			− 0.0011	0.0213		
Cardiovascular disease			− 0.0177	0.0518		
Cancer			0.0174	0.0327		
Depression			− 0.0440	0.0224*		
Multi-morbidity						
1 conditions					− 0.0204	0.0140
2 conditions					− 0.0426	0.0299
3 conditions					− 0.1497	0.2125
Constant	2.0319	0.2516	2.233	0.3515***	2.2193	0.2692
Observations	4160		2948		3632	
N participants	1247		1057		1174	
Rho	0.782		0.791		0.784	
BIC	− 6040.70		− 4100.96		− 5282.99	

This table reports the generalised least squares fixed effects regression with robust standard errors for three model specifications to estimate the association between BMI and EQ-5D-3L. The table reports analyses without imputation of missing values. A fixed-effects specification was selection based on the Hausman test. An alternative specification using the Tobit regression is provided in the supplementary material. Model specification 1 includes only BMI and age as variables. Model specification 2 and 3 include the effects of comorbidities. Rho indicates the fraction of variance due to the individual term. BIC Bayesian Information Criterion. Statistical significance at three thresholds are indicated by asterisks *** < 0.001 ** < 0.01 * < 0.05

In Table 4 we report the regression results for the secondary analysis in which changes in EQ-5D-3L are regressed against changes in BMI. A similar negative and statistically significant relationship between changes in BMI and changes in EQ-5D-3L was observed in model specifications 4–6 with an approximate magnitude of − 0.01 per unit change in BMI. Adding a covariate to adjust for asymmetry in the relationship between weight loss and EQ-5D-3L in model specification 5 estimated a coefficient of 0.0059. Adding together the coefficient for BMI and BMI change during weight loss implies that the relationship between BMI change and EQ-5D-3L changes is less during periods of weight loss (− 0.009) than weight gain (− 0.0146), however the difference was not statistically significant. The effect of changes in comorbidity was not associated with a significant change in EQ-5D-3L in model specification 6. The results of the regression analysis for changes in weight and changes in EQ-5D-3L are reported in the Supplementary Appendix Table S8 and report similar results to those described in Fig. 1.

**Table 4:** Regression estimates predicting change in EQ-5D-3L conditional on changes in BMI, including stratification by weight loss or weight gain

	Model specification 4: linear relationship		Model specification 5: asymmetric relationship		Model specification 6: multi-morbidity score	
	Coefficient estimate	Standard error	Coefficient estimate	Standard error	Coefficient Estimate	Standard error
Change in BMI	− 0.0111	0.0019***	− 0.0146	0.0041***	− 0.0173	0.0052***
Change in BMI during weight loss			0.0059	0.0057	0.0089	0.0072
Baseline EQ-5D	− 0.0878	0.0217***	− 0.0890	0.0218***	− 0.0840	0.0236***
12 week intervention	0.0052	0.0105	0.0049	0.0105	− 0.0059	0.0111
52 week intervention	− 0.0080	0.0086	− 0.0083	0.0086	− 0.010	0.0097
New comorbidity					− 0.0112	0.0138
Constant	0.0574	0.0196***	0.0633	0.0205***	0.0620	0.0222**
Observations	2616		2616		2093	

This table reports the ordinary least squares regression output for three model specifications to estimate the association between change in BMI and change in EQ-5D-3L. The table reports analyses without imputation of missing values. Model specification 4 includes change BMI and baseline BMI as variables. Model specification 5 includes an interaction term for change in BMI during periods of weight loss. Model specification 6 include the effects of comorbidities. Statistical significance at three thresholds are indicated by asterisks *** < 0.001 ** < 0.01 * < 0.05

Table 4 The regression analyses were stratified by time period (Tables S9–S12 in the Supplementary Appendix) and we observe some differences in the relationship between changes in BMI and changes in EQ-5D-3L between periods. However, across all time periods the results suggest there is a sizeable negative relationship between change in BMI and EQ-5D-3L, but the relationship did not always reach statistical significance.

## Discussion

This long-term follow-up of a RCT of a behavioural weight loss intervention provides estimates of the impact of BMI on HRQOL measured by the EQ-5D-3L. We have identified a significant negative relationship between BMI changes and HRQoL. These associations remained statistically significant after controlling for chronic diseases, age, and time-invariant factors; and was robust to alternative model specifications. Our analysis suggested that there may be a stronger relationship during periods of weight regain compared with weight loss, but the differences were not statistically significant in our adjusted regression analysis. A strong relationship between BMI and HRQoL would suggest that reducing obesity in the UK could improve health and well-being of the population over the long-term. These findings reinforce a need for effective policies and strategies to support individuals to lose weight, and avoid the trend for weight regain over the long-term. The estimated association between BMI and EQ-5D-3L could be used in economic evaluations to estimate QALYs for health economic analyses. In modelling studies it is necessary to extrapolate the long-term effects of an intervention on QALYs and these analyses will help to estimate short and long-term QALYs due to modified BMI trajectories to inform lifetime incremental cost-effectiveness calculations for new weight loss programmes.

Our findings support the findings from other longitudinal analyses of the relationship that changes in BMI have a statistically significant relationship with HRQoL [4, 5]. In a similar population of participants enrolling on a weight loss programme in the United States, the estimated relationship between BMI and EQ-5D-3L was reported to be − 0.0073 [12], which is lower than our estimated coefficient of − 0.011. However, their analysis included data collected after only 6 months. Our larger coefficient may be due to the longer follow-up of patients and inclusion of data during a period of weight regain. Comparing our analyses with minimally clinically important differences (MICD) in EQ-5D of 0.03 would suggest that weight loss of approximately − 7 kg are needed to achieve this threshold [25]. Whilst the relationship between BMI and quality of life is significant it will be challenging to detect a MICD from weight loss alone.

Studies of general population cohorts find differences in the strength of association with change in EQ-5D-3L between weight loss and weight regain. We identified a non-statistically significant difference in the relationships with change in EQ-5D-3L for people experiencing weight loss or weight regain. The reasons for the difference are not fully understood, but the motivation of participants to lose weight in this study may generate a positive response to weight loss compared to the general population. It is notable that 36% of participants in the WRAP trial reported a HRQoL of 1 on entry into the study so the non-symmetrical relationship between weight loss and weight gain may be due to a ceiling effect of the EQ-5D-3L, meaning that individuals experiencing weight loss cannot report improvements in HRQoL within the constructs of the EQ-5D-3L. Given that we did not identify a statistically significant difference between weight loss and weight regain we would recommend assuming a linear and symmetrical relationship between BMI and EQ-5D-3L, regardless of weight loss or regain, in modelling studies, particularly in individual patient simulations that account for the ceiling effect by design. Further research is needed to investigate the reasons for differences between these relationships.

It is interesting to note that in this analysis the effects of comorbid conditions did not have a significant impact on EQ-5D-3L. In contrast to previous analyses of cross-sectional data, these health conditions did not mediate the relationship between BMI and EQ-5D-3L [2]. We believe that this absence of association between comorbid conditions and HRQoL could be due to small sample size, missing data and duration of follow-up of this study. We detect significant association for depression and multi-morbidity index in analyses with multiple imputation. In neither case did the significant association with comorbid conditions alter the association between BMI and HRQoL Whilst we adjust for highly relevant comorbid conditions for this population, diabetes, cardiovascular disease and depression medications, there are a number of other health conditions, such as musculoskeletal complications or Chronic Obstructive Pulmonary Disease for which we do not have data.

Analysis of the relationship between BMI and EQ-5D-3L is particularly useful because the data include long-term follow-up of patients that are eligible to receive weight loss intervention. The trial recruited a larger proportion of White and less deprived participants than the English population. Mean EQ-5D-3L at baseline was 0.788, which is similar in magnitude to those reporting an health condition [26]. Therefore, the health burden of the sample is not substantially different to a population who would be eligible for weight loss intervention. As a consequence, the findings can be generalised and used in cost-utility analyses to estimate the QALY impacts of weight loss interventions. Estimating the impact of weight changes on HRQoL from intervention studies is challenging because it is not possible to exclude the confounding benefits of receiving an intervention, and participating in a trial. We assume that change in BMI is associated with change in HRQoL, whereas other factors associated with the intervention, such as socialisation, may also bias this association. The long duration of follow-up within the trial and brief intervention arm will help to mitigate this effect by looking at the relationship between BMI and HRQoL over a sustained period after the intervention was delivered. Furthermore, covariates to adjust for the effects of the 12-week and 52-week intervention were non-significant throughout this analysis, indicating that participation in the intervention did not provide additional improvements in HRQoL once changes in BMI were accounted for. However, further research is needed to explore how the components of the intervention impact HRQoL independently of BMI.

Another challenge when estimating EQ5D is that it does not display a normal distribution. The distribution of EQ5D is limited at both ends and has gaps where values are not feasible. Mixture models [27, 28] have been shown to outperform standard regression techniques in predicting EQ5D [29, 30]; however, these models were developed specifically for utility mapping and are not currently possible within a panel data framework. Given that we find very similar results using the generalised least squares fixed effects model estimating EQ5D-3L and the linear model estimating the change in EQ5D-3L (which is normally distributed) as well as the random effects Tobit model predicting EQ5D-3L (see supplementary material), we believe that our results are sufficiently robust in predicting mean change in EQ5D-3L.

We have conducted panel regression analyses and within subject analyses to address confounding due to time-invariant heterogeneity, however the analysis may be confounded by time-varying factors and may have biased the effect estimates. Therefore, it is not possible to conclude that the changes in BMI caused the changes in EQ-5D-3L.

## Conclusion

We identified a significant negative relationship between BMI and EQ-5D-3L over 5 years in a RCT of a group-based weight loss interventions. This evidence provides a strong rationale for investment in obesity prevention, weight loss services and other interventions to support weight maintenance.

## Supplementary Information

Below is the link to the electronic supplementary material.

Supplementary file1 (DOCX 36 KB)
