# Supplementary material for: Estimating the impact of changes in weight and BMI on EQ-5D-3L: a longitudinal analysis of a behavioural group-based weight loss intervention
Source: Qual Life Res. 2022 Jul 7;31(11):3283–92. doi: 10.1007/s11136-022-03178-z (PMC9546944; doi:10.1007/s11136-022-03178-z)
Supplement: Supplementary file 1 — Supplementary file1 (DOCX 36 KB) [file 11136_2022_3178_MOESM1_ESM.docx]

Supplementary Appendix

## Wooldridge (2010) test for Attrition

Table S1: Results of the Wooldridge test for attrition in the data

|  |  | Coefficient | T-test pvalue | Wald p-value |
| --- | --- | --- | --- | --- |
| Step 4 – Mills only | Mills | .014926 | 0.674 |  |
| Step 5 – With interaction | Mills | -.1111906 | 0.416 | 0.1986 |
|  | Mills_month3 | -.0319969 | 0.270 |  |
|  | Mills_year1 | .153808 | 0.275 |  |
|  | Mills_year2 | .1552463 | 0.265 |  |
|  | Mills_year5 | -.013685 | 0.926 |  |

## Missing Data

Table S2: Summary of missing data for main variables by timepoint

|  | Baseline | 3 month | 1 year | 2 year | 5 year |
| --- | --- | --- | --- | --- | --- |
| BMI | 0 | 263 | 444 | 411 | 451 |
| EQ-5D | 58 | 347 | 519 | 524 | 703 |
| Diabetes | 137 | 137 | 574 | 719 | 359 |
| Depression | 396 | 643 | 741 | 719 | 450 |

## Supplementary Results

Table S3: Regression estimates predicting EQ-5D conditional on weight and other associated factors, a fixed effects model with robust standard errors without imputation of missing weight and EQ-5D

|  | No Co-morbidities | | Independent covariate | | Multi-morbidity score | |
| --- | --- | --- | --- | --- | --- | --- |
|  | Coefficient estimate | Standard error | Coefficient estimate | Standard error | Coefficient estimate | Standard error |
| Weight | -.0039225 | .0005581*** | -.0045099 | .0007586*** | -.004041 | .0006267*** |
| Age | -.015766 | .0082076 | -.020111 | .0112587 | -.0228161 | .0086782*** |
| Age-squared | .0000362 | .000068 | .0000648 | .0000916 | .0000957 | .0000719 |
| Diabetes |  |  | .0006801 | .0211581 |  |  |
| Cardiovascular disease |  |  | -.0168751 | .0511962 |  |  |
| Cancer |  |  | .0163091 | .0326299 |  |  |
| Depression |  |  | -.0478277 | .0224574 |  |  |
| Multi-morbidity |  |  |  |  |  |  |
| 1 conditions |  |  |  |  | -.0224879 | 0140652 |
| 2 conditions |  |  |  |  | -.0435607 | .0297194 |
| 3 conditions |  |  |  |  | -.1531583 | .2155086 |
| Constant | 1.912079 | .2497838*** | 2.12049 | .3534104*** | 2.128067 | .2695916*** |
| Observations | 4168 |  | 2951 |  | 3635 |  |
| N participants | 1247 |  | 1058 |  | 1175 |  |
| Rho | 0.7819 |  | 0.7771 |  | 0.7847 |  |
| BIC | -6032.13 |  | -4058.94 |  | -5268.00 |  |
| *** <0.001 **<0.01 *<0.05 | | | | | | |

Table S4: Regression estimates predicting EQ-5D conditional on BMI and other associated factor including statin use and hypertension medication, a fixed effects model with robust standard errors

|  | No Co-morbidities | | Independent covariate | | Multi-morbidity score | |
| --- | --- | --- | --- | --- | --- | --- |
|  | Coefficient estimate | Standard error | Coefficient estimate | Standard error | Coefficient estimate | Standard error |
| BMI | -0.0107 | 0.0016 *** | -0.01220 | .0021277*** | -.011264 | .0018796*** |
| Age | -0.0206 | 0.0082 ** | -0.0247121 | .0111716* | -.0260371 | .0087809** |
| Age-squared | 0.0001 | 0.0001 | .0001009 | .0000916 | .0001214 | .0000728 |
| Diabetes |  |  | .000549 | .0212027 |  |  |
| Cardiovascular disease |  |  | -.017353 | .051904 |  |  |
| Cancer |  |  | .0167115 | .0328386 |  |  |
| Depression |  |  | -.0451987 | .0224328 *** |  |  |
| Hypertension |  |  | .0211189 | .0224397 |  |  |
| Statin use |  |  | .0104822 | .0192922 |  |  |
| Multi-morbidity |  |  |  |  |  |  |
| 1 conditions |  |  |  |  | -.0007985 | .0136416 |
| 2 conditions |  |  |  |  | -.0254939 | .0166726 |
| 3 conditions |  |  |  |  | -.0262905 | .0248431 |
| 4 conditions |  |  |  |  | -.0248654 | .0415953 |
| 5 conditions |  |  |  |  | .1572596 | .0278926 |
| Constant | 2.0319 | 0.2516 | 2.236267 | .3511906 *** | 1.432658 | .2713455 |
| Observations | 4160 |  | 2948 |  | 5409 |  |
| N participants | 1247 |  | 1057 |  | 1267 |  |
| Rho | 0.782 |  | .77772252 |  | .50450511 |  |
| *** <0.001 **<0.01 *<0.05 | | | | | | |

Table S5: Regression estimates predicting EQ-5D conditional on BMI and other associated factors, a fixed effects model with robust standard errors with multiple imputation of missing BMI and EQ-5D

|  | No Co-morbidities | | Independent covariate | | Multi-morbidity score | |
| --- | --- | --- | --- | --- | --- | --- |
|  | Coefficient estimate | Standard error | Coefficient estimate | Standard error | Coefficient estimate | Standard error |
| BMI | -.0111618 | .0018854*** | -.010707 | .0018569*** | -.0104974 | .0018796*** |
| Age | -.0018389 | .008892 | -.0026428 | .0087481 | -.0041327 | .0087809 |
| Age-squared | -.0000462 | .0000735 | -.0000345 | .0000729 | -.0000144 | .0000728 |
| Diabetes |  |  | -.003849 | .0186844 |  |  |
| Cardiovascular disease |  |  | -.0146174 | .0477909 |  |  |
| Cancer |  |  | .0039927 | .0465445 |  |  |
| Depression |  |  | -.0908124 | .0142532*** |  |  |
| Multi-morbidity |  |  |  |  |  |  |
| 1 conditions |  |  |  |  | -.0567712 | .0115936*** |
| 2 conditions |  |  |  |  | -.1042786 | .0295771*** |
| 3 conditions |  |  |  |  | -.130906 | .1867788 |
| Constant | 1.409741 | .2683711*** | 1.421758 | .2639833*** | 1.432658 | .2653901 |
| Observations | 5409 |  | 5409 |  | 5409 |  |
| N participants | 1267 |  | 1267 |  | 1267 |  |
| Rho | .523618 |  | .50164158 |  | .50450511 |  |
| *** <0.001 **<0.01 *<0.05 | | | | | | |

Table S6: Regression estimates predicting EQ-5D conditional on BMI and other associated factors, a fixed effects model with robust standard errors restricted to participants who attended the follow-up at 5 years

|  | No Co-morbidities | | Independent covariate | | Multi-morbidity score | |
| --- | --- | --- | --- | --- | --- | --- |
|  | Coefficient estimate | Standard error | Coefficient estimate | Standard error | Coefficient estimate | Standard error |
| BMI | -.010953 | .0017669*** | -.0125675 | .0022959*** | -.0115672 | .0019181 *** |
| Age | -.0217719 | .0084745** | -.0257298 | .0114519* | -.0264456 | .0087834 |
| Age-squared | .0000885 | .0000701 | .0001138 | .0000935 | .0001283 | .0000729 |
| Diabetes |  |  | .0002684 | .0236041 |  |  |
| Cardiovascular disease |  |  | -.0189779 | .0520736 |  |  |
| Cancer |  |  | .0163357 | .0327127 |  |  |
| Depression |  |  | -.0389888 | .0249485 |  |  |
| Multi-morbidity |  |  |  |  |  |  |
| 1 conditions |  |  |  |  | -.0185196 | .0153616 |
| 2 conditions |  |  |  |  | -.0379902 | .0319088 |
| 3 conditions |  |  |  |  | -.1467347 | .2143078 |
| Constant | 2.090595 | .2626279*** | 2.288329 | .3621297 *** | 2.247766 | .2766436*** |
| Observations | 3265 |  | 2366 |  | 2871 |  |
| N participants | 814 |  | 738 |  | 787 |  |
| Rho | .76272345 |  | .75505728 |  | .75999103 |  |
| *** <0.001 **<0.01 *<0.05 | | | | | | |

Table S7: Estimated average marginal effects predicting EQ-5D conditional on BMI and other associated factors, a tobit random effects model robust standard errors with multiple imputation of missing BMI and EQ-5D

|  | No Co-morbidities | | Independent covariate | | Multi-morbidity score | |
| --- | --- | --- | --- | --- | --- | --- |
|  | Average marginal effects | Standard error | Average marginal effects | Standard error | Average marginal effects | Standard error |
| BMI | -.0108114 | .00085*** | -.0103041 | .00103*** | -.0099238 | .00091*** |
| Age | -.0002405 | .00260 | .0028911 | .00319 | -.0004155 | .00282 |
| Age-squared | -.0000332 | .00002 | -.0000534 | .00003 | -.0000264 | .00003 |
| Diabetes |  |  | -.0027704 | .01352 |  |  |
| Cardiovascular disease |  |  | -.0103958 | .03377 |  |  |
| Cancer |  |  | -.0172763 | .03466 |  |  |
| Depression |  |  | -.1151859 | .01208*** |  |  |
| Multi-morbidity |  |  |  |  |  |  |
| 1 conditions |  |  |  |  | -.0635377 | .00953*** |
| 2 conditions |  |  |  |  | -.1114256 | .02214*** |
| 3 conditions |  |  |  |  | -.222135 | .13090 |
| Constant | 1.647744 |  |  |  |  |  |
| Observations | 4,160 |  | 2,948 |  | 3,632 |  |
| N participants | 1,247 |  | 1,057 |  | 1,174 |  |
| Rho | .7042301 |  | .6694691 |  | .6915871 |  |
| *** <0.001 **<0.01 *<0.05 | | | | | | |

Table S8: Regression estimates predicting change in EQ-5D conditional on changes in weight with an interaction term for periods of weight loss, a random effects model without imputation of missing data

|  | Linear relationship | | Asymmetric relationship | | Multi-morbidity score | |
| --- | --- | --- | --- | --- | --- | --- |
|  | Coefficient estimate | Standard error | Coefficient Estimate | Standard error | Coefficient Estimate | Standard error |
| Change in weight | -.0039893 | .0007044 *** | -.0057 | .0015*** | -.0070 | .0019*** |
| Change in weight during weight loss |  |  | .0028 | .0021 | .0043 | .0026 |
| Baseline EQ-5D | -.0863999 | .0216*** | -.0876 | .0216*** | -.0867 | .0235*** |
| 12 week intervention | -.0058 | .0105 | -.0053 | .0105 | -.0062 | .0112 |
| 52 week intervention | -.0073 | .0086 | -.0078 | .0086 | -.0098 | .0097 |
| New comorbidity |  |  |  |  | -.0135 | .0139 |
| Constant | .0556 | .0195** | .0628 | .0202** | .0631 | .0220** |
| Observations | 2620 |  | 2620 |  | 2094 |  |

Table S9: Regression estimates predicting change in EQ-5D conditional on changes in BMI between baseline and 3 months, an ordinary least squares model without imputation of missing data

|  | Linear relationship | | Asymmetric relationship | | Multi-morbidity score | |
| --- | --- | --- | --- | --- | --- | --- |
|  | Coefficient estimate | Standard error | Coefficient Estimate | Standard error | Coefficient Estimate | Standard error |
| Change in BMI | -.0085478 | .0038534* | -.0086352 | .0142025 | -.0100374 | .0155688 |
| Change in BMI during weight loss |  |  | .0000975 | .0156016 | -.0005704 | .0170702 |
| Baseline EQ-5D | -.1990933 | .0346093*** | -.199096 | .034676*** | -.1879938 | .036588 |
| 12 week intervention | .0105339 | .0173355 | .0105385 | .0173181 | .0123252 | .0178847 |
| 52 week intervention | .0002561 | .0172037 | .0002585 | .0171853 | -.0029039 | .0179345 |
| New comorbidity |  |  |  |  | -.0748193 | .0322583 |
| Constant | .1456491 | .0347517*** | .1456701 | .0354021*** | .1332814 | .0371545 |
| Observations | 883 |  | 883 |  | 802 |  |

Table S10: Regression estimates predicting change in EQ-5D conditional on changes in BMI between 3 months and 12 months, an ordinary least squares model without imputation of missing data

|  | Linear relationship | | Asymmetric relationship | | Multi-morbidity score | |
| --- | --- | --- | --- | --- | --- | --- |
|  | Coefficient estimate | Standard error | Coefficient Estimate | Standard error | Coefficient Estimate | Standard error |
| Change in BMI | -.0126899 | .00316*** | -.0347316 | .0090914*** | -.0402923 | .0093968*** |
| Change in BMI during weight loss |  |  | .0303357 | .0110727** | .041066 | .0117312** |
| Baseline EQ-5D | -.0492589 | .0366256 | -.0536354 | .0362043 | -.0349476 | .0382704 |
| 52 week intervention | -.0187821 | .0136278 | -.0182335 | .0135418 | -.0096345 | .0143521 |
| New comorbidity |  |  |  |  | -.0499464 | .0222252 |
| Constant | .0311221 | .0331221 | .0551857 | .0331751 | .0416395 | .0347009 |
| Observations | 654 |  | 654 |  | 802 |  |

Table S11: Regression estimates predicting change in EQ-5D conditional on changes in BMI between 12 months and 24 months, an ordinary least squares model without imputation of missing data

|  | Linear relationship | | Asymmetric relationship | | Multi-morbidity score | |
| --- | --- | --- | --- | --- | --- | --- |
|  | Coefficient estimate | Standard error | Coefficient Estimate | Standard error | Coefficient Estimate | Standard error |
| Change in BMI | -.0051288 | .004437 | -.0060967 | .0073039 | -.0127359 | .0091479 |
| Change in BMI during weight loss |  |  | .0025769 | .0115537 | .0060741 | .01374 |
| Baseline EQ-5D | -.0114966 | .0454527 | -.0123995 | .0462575 | -.0297833 | .0527576 |
| New comorbidity |  |  |  |  | .0289961 | .0391737 |
| Constant | -.0006003 | .0394418 | .0017086 | .0425787 | .0219941 | .048324 |
| Observations | 606 |  | 606 |  | 404 |  |

Table S12: Regression estimates predicting change in EQ-5D conditional on changes in BMI between 24 months and 60 months, an ordinary least squares model without imputation of missing data

|  | Linear relationship | | Asymmetric relationship | | Multi-morbidity score | |
| --- | --- | --- | --- | --- | --- | --- |
|  | Coefficient estimate | Standard error | Coefficient Estimate | Standard error | Coefficient Estimate | Standard error |
| Change in BMI | -.0141776 | .0036893*** | -.0113142 | .0063081 | -.0085062 | .0093483 |
| Change in BMI during weight loss |  |  | -.0054561 | .0105102 | -.00983 | .0145668 |
| Baseline EQ-5D | -.0180882 | .0654184 | -.0142631 | .0672411 | .0233002 | .0788636 |
| New comorbidity |  |  |  |  | .0275252 | .0231056 |
| Constant | -.0100208 | .0583003 | -.0188504 | .0425787 | -.0514079 | .0745132 |
| Observations | 473 |  | 473 |  | 336 |  |
